# Benzo[a]pyrene and UV light co-exposure: differential effects on oxidative stress and genotoxicity in human keratinocytes and ex vivo skin

**DOI:** 10.1007/s00204-025-04098-w

**Published:** 2025-07-01

**Authors:** Christian Kersch, Viktor Masutin, Rasha Alsaleh, Simone Schmitz-Spanke

**Affiliations:** Institute and Outpatient Clinic of Occupational, Social, and Environmental Medicine, Friedrich-Alexander-University of Erlangen-Nuremberg, Henkestr. 9–11, 91054 Erlangen, Germany

**Keywords:** Benzo(a)pyrene, UV irradiation, Keratinocytes, Human ex vivo skin, Genotoxicity, Combined effects

## Abstract

Human skin exposure to environmental/occupational UV irradiation and polycyclic aromatic hydrocarbons (PAHs) is a concern. This study investigated the impact of the combined exposure on metabolism, cytotoxicity/viability, oxidative stress, and genotoxicity in human skin models. To understand the dose–response relationship, we exposed our models to a range of benzo[a]pyrene (B[a]P) concentrations. For the combined exposure scenario, the models were treated with B[a]P followed by UV irradiation. Furthermore, we employed two in vitro models: human keratinocytes and human skin explants. This allowed us to assess potential model-dependent variations in the observed toxicological effects. Keratinocytes displayed a partially synergistic response in most measured parameters at 0.4 µM B[a]P or higher after combined exposure. The comet assay identified significant DNA damage solely due to UV irradiation, and this damage was not further increased by combined exposure. Conversely, H2AX phosphorylation showed a dose-dependent increase in response to the combined exposure. Human skin explants displayed minimal changes in oxidative stress markers compared to keratinocytes. Additionally, DNA damage in explants was significantly reduced following UV irradiation alone and remained lower than after single B[a]P exposure, even with combined exposure. These findings suggest a potential UV-induced protective mechanism in ex vivo human skin under these conditions. Furthermore, they highlight the differential response of the models, with explants exhibiting lower reactivity due to their inherent complexity and robustness, potentially reflecting the protective mechanisms present in live skin.

**Supplementary Information:**

The online version contains supplementary material available at 10.1007/s00204-025-04098-w.

## Introduction

Polycyclic aromatic hydrocarbons (PAHs) are ubiquitous environmental pollutants formed primarily from incomplete combustion of organic materials. They also contribute significantly to occupational exposure, particularly for outdoor workers. While the most concerning consequence of PAH exposure is cancer, with links to lung, skin, and bladder cancers at high exposure levels (Boffetta et al. 1997), the focus of this study is on skin cancer. Unlike PAHs, which require metabolic activation (primarily by cytochrome P450;) or light absorption to exert their toxic effects (Fu et al. 2012; Reed et al. 2018), UV irradiation itself is the primary culprit in skin cancer development. Notably, Germany recognized UV irradiation-induced skin cancer as an occupational disease in 2015 (Diepgen et al. 2015).

Skin cancer remains a significant public health concern due to its high incidence and the widespread environmental presence of PAHs and UV irradiation. Understanding the potential interactive effects of these exposures is crucial for effective risk assessment and prevention strategies. While the independent carcinogenic effects of PAHs and UV irradiation are well-established, the evidence for a combined, synergistic effect on human skin cancer development remains inconclusive. A systematic review did not identify compelling evidence for synergy in squamous cell carcinoma development (Weistenhofer et al. 2022). Conversely, animal studies have yielded conflicting results, with reports of both synergistic (Stenback et al. 1973; Wang et al. 2005) and non-synergistic effects on tumor development (Teutschlaender 1937). Interestingly, Stenback et al. observed that increasing UV irradiation intensity could even reverse the synergistic effect.

This complexity extends to the timing of exposure. In mice, the application of 7,12-dimethylbenz[a]anthracene (DMBA) one hour prior to irradiation resulted in fewer tumors compared to the reversed order (Stenback 1975). This finding contrasts with human keratinocyte studies, where the opposite sequence exhibited a stronger tumor-suppressive effect (von Koschembahr et al. 2020b). These discrepancies suggest that the interplay between PAHs and UV irradiation might be highly dependent on the specific exposure scenario and cell type, potentially due to variations in DNA repair mechanisms.

While a definitive demonstration of a synergistic carcinogenic effect from combined UV irradiation and PAH exposure is still lacking, the underlying mechanisms suggest such an interaction might be plausible. Supporting evidence comes from animal, in vitro, and ex vivo studies. One proposed mechanism involves the enhanced metabolic conversion of B[a]P to its potent carcinogen, diol epoxide, under UV irradiation (Burke and Wei 2009). Additionally, oxidative stress is thought to play a significant role (Burke and Wei 2009; Saladi et al. 2003). Studies using cell lines and ex vivo human skin models have delved deeper into the oxidative stress hypothesis (Botta et al. 2009; von Koschembahr et al. 2018, 2020b; Xia et al. 2015).

Previous studies investigating the combined effects of PAHs and UV irradiation often employed a limited concentration range, hindering a complete understanding of the transition from cellular adaptation to adverse effects. To overcome this limitation, our study utilized a broad range of B[a]P concentrations and monitored a panel of endpoints encompassing various cellular responses. These responses included metabolism, oxidative stress, and genotoxicity, providing a comprehensive picture of cellular behavior. Additionally, we employed two distinct models, human keratinocytes (KeratinoSens cells) and human skin explants, to evaluate potential model-dependent effects. The KeratinoSens cell line was specifically chosen as it provides a well-established model for assessing the activation of the Keap1-NRF2-ARE pathway, which plays a role in the anticipated oxidative stress response. Both models received single exposures to UVA/UVB irradiation or varying B[a]P concentrations. For the combined exposure scenario, cells or explants were treated with B[a]P followed by UV irradiation. Our findings underscore the ex vivo human skin model as the more representative reflection of human skin physiology. The reduced reactivity of ex vivo skin, attributed to its robustness and complexity, necessitates cautious interpretation of in vitro data when extrapolating to human responses.

## Materials and methods

### Chemicals and reagents

Standard chemicals for cell culture were obtained from c.c.pro GmbH (Oberdorla, Germany). B[a]P was purchased from Merck KGaA, (Darmstadt, Germany, CAS: 50-32-8); and dimethyl sulfoxide (DMSO) from AppliChem GmbH (Darmstadt, Germany, CAS: 67–68-5). Other chemicals were also obtained from Alfa Aesar by Thermo Fisher GmbH (Kandel, Germany); Merck KGaA (Darmstadt, Germany); Life Technologies by Thermo Fisher Scientific (Waltham, USA); Promega GmbH (Walldorf, Germany); Santa Cruz Biotechnology, Inc. (Heidelberg, Germany); and Carl Roth GmbH + Co. KG (Karlsruhe, Germany).

### Cell culture

KeratinoSens cells (obtained from Givaudan, Vernier, Switzerland) were cultivated as described (De Rentiis et al. 2021). A consistent seeding density of 100,000 cells/mL was employed across all plate formats (96-well, 6-well, etc.). In the case of 96-well plates, each well received a 100 µL aliquot of the cell suspension, resulting in a seeding of 10,000 cells per well. For details see Supplementary Methods.

### Skin preparation

Human skin samples were obtained from the university hospital following abdominal plastic surgery procedures. The study adhered to the university’s ethical guidelines, and patients provided written consent. Skin explants were incubated for 24 h before further experimentation (see Supplementary Methods).

### Treatment with B[a]P and UV irradiation

#### Keratinocytes

KeratinoSens cells were exposed to 8 concentrations of B[a]P (0.000004–40 μM, dissolved in DMSO). The dose range of B[a]P was determined in preliminary tests using the Neutral Red assay (see Fig. S1 and Supplementary Results for details). Untreated controls were incubated with pure medium containing the same amount of DMSO as with the treated cells (< 1% DMSO).

Without UV irradiation, cells were exposed to B[a]P for 24 h each. For irradiation, cells were incubated with medium containing B[a]P and were directly exposed to UV (room temperature at a dose of 3.5 J/cm^2^ with 95% UVA and 5% UVB (0.97 mW/cm^2^) see Supplementary Data for details). Afterwards, cells were further incubated until 24 h (no medium change). Irradiation was carried out with a UV LED chamber BS-02 UV/VIS (Opsytec Dr. Gröbel GmbH, Ettlingen, Germany) which emits wavelengths in the 280–400 nm range.

#### Skin explants

B[a]P was topically applied to the skin at concentrations of 0.318, 31.8, and 318 ng/cm^2^ (equivalent of B[a]P amount per cm^2^ compared to cell culture doses 0.004, 0.4 and 4 µM in cell culture, see Supplementary Information for further details). This was achieved by applying 10 µL of a solution containing the appropriate B[a]P concentration. UV irradiations were performed using the abovementioned UV LED chamber (7 J/cm^2^ with 95% UVA, 5% UVB). See Supplementary Information for details.

### Assessment of CYP and NQO1 activity

Enzyme activity was measured as previously described, with the following modifications: the assays were adapted for keratinocytes and skin, and 7-ethoxyresorufin-O-deethylase (7-EROD) was used instead of 7-benzyloxyresorufin-O-debenzylase (BROD) to detect CYP activity (Pink et al. 2017). The following steps were identical for cells and skin homogenates. 100 µL of the reaction mixture (CYP: 10 μM 7-ethoxyresorufin [stock solution of 10 mM in DMSO, then as a working solution in PBS containing 0.1% Triton X-100]; NQO1: 25 mM TRIS (pH 7.4), 0.4 mM NADH, 0.1% Triton X-100) were added to each well. The plates were incubated for 5 min (37 °C, 5% CO_2_), then the necessary cofactors and detection reagents (CYP: 50 μL of a solution of NADPH [1 mM in water]; NQO1: 20 µL dichlorophenolindophenol [100 mM stock solution in DMSO, diluted to 1.5 mM in water]) were added. After 10 min of incubation (37 °C, 5% CO_2_), the fluorescence for CYP (at λ(ex/em) 544/595) or absorbance for NQO1 (at 600 nm) was measured, respectively. Keratinocyte samples comprised 5 biological and 8 technical replicates, while ex vivo samples included 3 biological and 4 technical replicates.

### Assessment of cytotoxicity, metabolic activity, and mitochondrial function

Lactate dehydrogenase (LDH) release was measured in both keratinocytes and skin explants. Metabolic activity was evaluated using the enzymatic conversion of MTT (3-(4,5-dimethylthiazol-2-yl)−2,5-diphenyltetrazolium bromide) in both models. Mitochondrial membrane potential (MMP) was measured only in keratinocytes. The MTT and MMP assays were performed as previously described (Pink et al. 2017). See Supplementary Data for details.

The LDH activity was determined in the medium by reducing iodonitrotetrazolium chloride (INT) into a formazan. Briefly, 50 µL of medium (supernatant) and 50 µL of each reaction solution (200 mM TRIS pH 8; 50 mM sodium lactate; and PMS (phenazine methosulfate)/INT/NAD solution were transferred into a new 96-well plate. The latter is prepared by mixing 100 µL of 29 mM PMS in water, 100 µL of 65 mM INT in DMSO, and 2.3 mL of 5 mM NAD^+^ dissolved in water) were transferred into a new 96-well plate. After incubation at room temperature for 5 min, the absorbance was measured at 490 nm. For keratinocyte MTT assays, *n* = 5 biological replicates and *n* = 8 technical replicates were used. For keratinocyte LDH assays, *n* = 5 biological replicates and *n* = 4 technical replicates were used. For ex vivo samples, *n* = 3 biological replicates and *n* = 4 technical replicates were used for both MTT and LDH assays.

### Assessment of oxidative stress

Levels of reactive oxygen species (ROS) were quantified using the fluorescent dye 2′,7′-dichloro-dihydrofluorescein diacetate (H_2_DCF-DA). Following PBS washing, cells were incubated with 20 μM H_2_DCF-DA for 30 min at 37 °C and 5% CO_2_. Cells were then washed three times with PBS, and fluorescence was measured after adding 100 μL PBS at λ(ex/em) 485/535 nm.

Reduced glutathione (GSH) and oxidized glutathione (GSSG) levels were determined to evaluate the antioxidant capacity of keratinocytes and skin. Briefly, for measuring GSH levels 100 μL reaction mixture containing PBS supplemented with 5 mM EDTA, 1 mM DTNB (5,5′-Dithiobis-2-nitrobenzoic acid), 1 U/mL GSSG reductase, and 0.2% Triton X-100 was added to each well and incubated for 30 s at room temperature. Subsequently, 20 μL of the activation solution (NADPH in water, final concentration of 0.25 mM) was added, and absorbance was immediately measured at 415 nm. To measure GSSG levels, an inhibitor solution (3 mM 1-methyl-2-vinylpyridinium triflate and 0.2% Triton X-100 in water) was added for two minutes at room temperature before proceeding as described for GSH.

A byproduct of lipid peroxidation is malondialdehyde (MDA), which reacts with 1-methyl-2 phenylindole to the chromophore. 100 µL 1-methyl-2-phenylindole were added (10 mM in 25% methanol in acetonitrile) to 96-well plates or 50 µL of the skin homogenate followed by 20 µL of HCl, 37%. After incubation for two hours at 45 °C, absorbance was read out at 595 nm.

The luciferase gene induction in KeratinoSens cells reflects Keap1-Nrf2-ARE pathway activation. The cells were washed once with PBS. Following a 20-min incubation with cell lysis buffer (passive 1 × lysis buffer; Promega GmbH, Walldorf, Germany), luminescence was measured using a modified luciferase substrate (30 mM tricine pH 7.8, 4 mM MgSO4, 0.15 mM EDTA, 0.1875 mM ATP, 30 mM DTT (Dithiothreitol), and 0.15 mM luciferin).

All endpoints were determined in keratinocytes, while glutathione and lipid peroxidation were additionally assessed in ex vivo skin. For keratinocytes, *n* = 5 biological replicates and *n* = 8 technical replicates were used. For ex vivo skin samples, *n* = 3 biological replicates and *n* = 4 technical replicates were used.

### Determination of genotoxicity

Phosphorylation of histone H2AX (γH2AX) was quantified using a Promega LUMIT immunoassay cellular-system assay kit, employing a combination of rabbit anti-histone H2AX and mouse anti-phospho-histone H2AX monoclonal antibodies. Briefly, 40 µL of pre-treated cell suspension or skin homogenate were incubated with 10 µL of 0.1% digitonin solution for 20 min in 96-well plates. An antibody mix containing 15 ng/well each of the primary, LgBiT anti-mouse, and SmBiT anti-rabbit antibodies was added for 60 min. Finally, 10 µL of the detection reagent was added, and the luminescence signal was integrated for 1 s per well. This assay was carried out using two biological replicates and four technical replicates in both models.

In addition, we conducted a comet assay in keratinocytes. A detailed description of the comet assay is provided in the Supplementary Data.

### Calculation of the benchmark dose

A benchmark-dose (BMD) calculation of the endpoints measured in keratinocytes was implemented, using Proast (version 70.1; https://proastweb.rivm.nl/). Modeling was performed with a benchmark response (BMR) of 0.05 and a 95% confidence interval. The model with the lowest AIC (Akaike information criterion) was selected and the BMD, BMDU (upper confidence interval), BMDL (lower confidence interval), and model were reported for all endpoints of combined exposure (B[a]P + UV) that permitted such calculation.

### Statistical analysis

The data are presented as the mean ± SD. Analysis of variance (ANOVA) was used to evaluate the differences between groups (Origin 2019). Statistical significance is defined as any p-value less than *p* < 0.05 and is represented in the graphs by asterisks (* = *p* < 0.05; ** = *p* < 0.01; *** = *p* < 0.001).

### Additional measurements and calculations

Measurement of B[a]P uptake and metabolism as a function of time, B[a]P dose, and UV irradiation was performed using fluorescence spectroscopy. A simple synergy calculation was also performed Methods and results are detailed in the Supplementary Data.

## Results

### Combined exposure had a significantly greater impact on CYP activity compared to single exposure in both models

We began our investigations by examining CYP activity in both keratinocytes and human skin explants. We measured the activity using the 7-EROD assay. While this assay is highly specific for CYP1 A1, it also detects CYP1 A2 activity to a lesser extent, and CYP1B1 minimally (Yu et al. 2001). Our focus was on CYP1 A1 activity, as it was initially considered the primary enzyme responsible for B[a]P activation. However, emerging evidence also suggests a role for CYP1B1 in the metabolism of B[α]P and 7,8-diol (Kim et al. 1998; Shimada and Fujii-Kuriyama 2004).

**Fig. 1:**
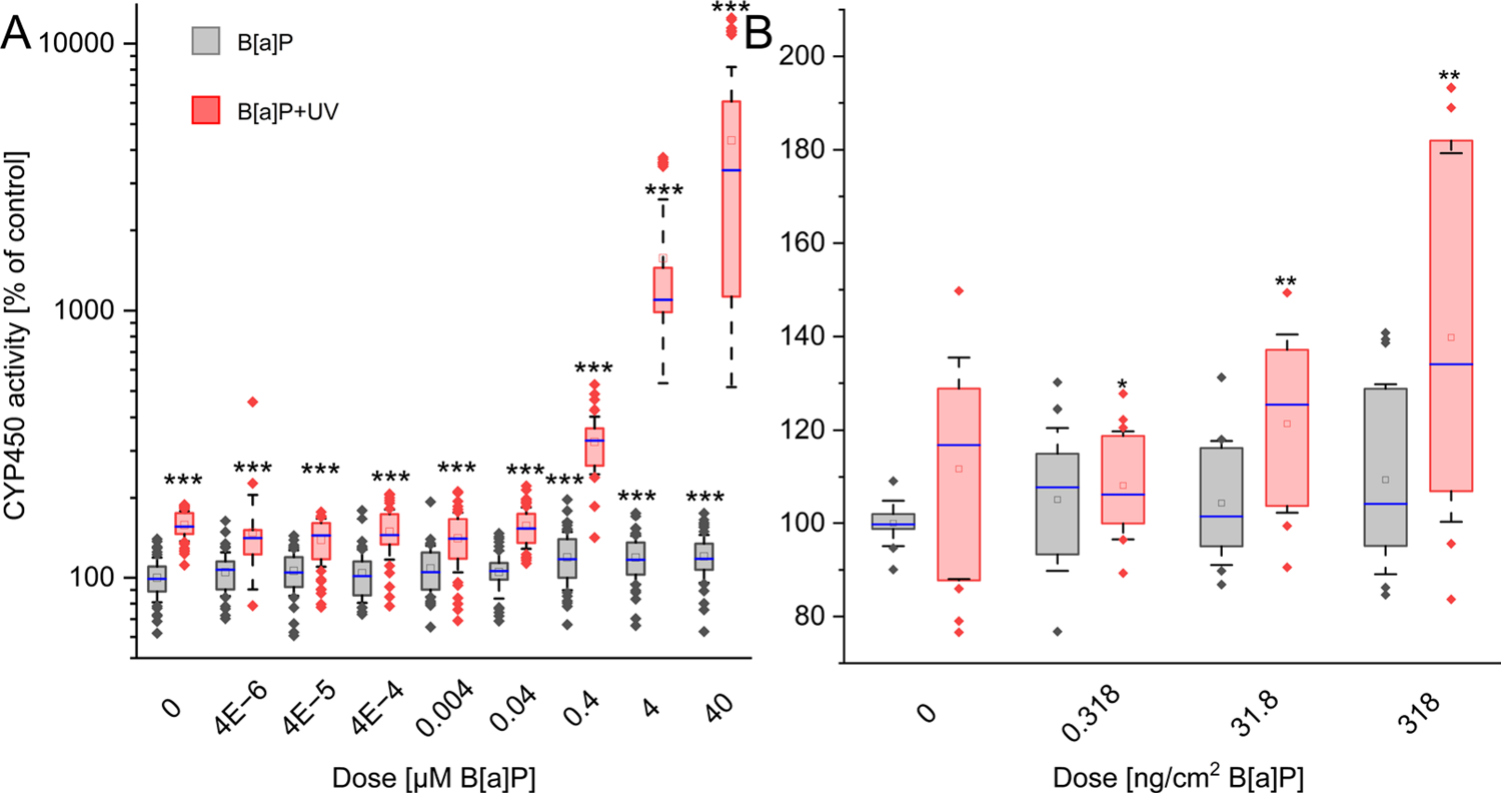
Relative changes of the dose-dependent activity of CYP450 in both models. **A** Cells or **B** skin explants were exposed for 24 h either to B[a]P or UV alone or in combination at the start of exposure. Results represent the mean ± SD. Statistical significance denotes a comparison to control condition without UV irradiation (* = *p* < 0.05; ** = *p* < 0.01; *** = *p* < 0.001). *n* = (keratinocytes: 5 biological replicates, 8 technical replicates; ex vivo: 3 biological replicates, 4 technical replicates) See Supplementary Excel Datasheet for further information. Please note the logarithmic representation of CYP activity on the Y-axis

In our models, single B[a]P exposure induced only a minor effect, while single UV irradiation elicited a significant increase in CYP activity in keratinocytes. The combined exposure to UV irradiation and B[a]P resulted in a pronounced dose-dependent increase in CYP450 activity in keratinocytes starting from 0.4 µM B[a]P (Fig. 1A). In ex vivo skin, the increase was also dose-dependent but less pronounced (Fig. 1B).

### UV irradiation drives viability impairment in combined B[a]P exposure, with enhanced susceptibility in keratinocytes

We employed three distinct assays to assess cytotoxicity and/or viability in both models. The integrity of cellular membranes (LDH) and overall metabolic activity (MTT) were measured in both models, while mitochondrial function (MMP) was specifically evaluated in keratinocytes.

**Fig. 2:**
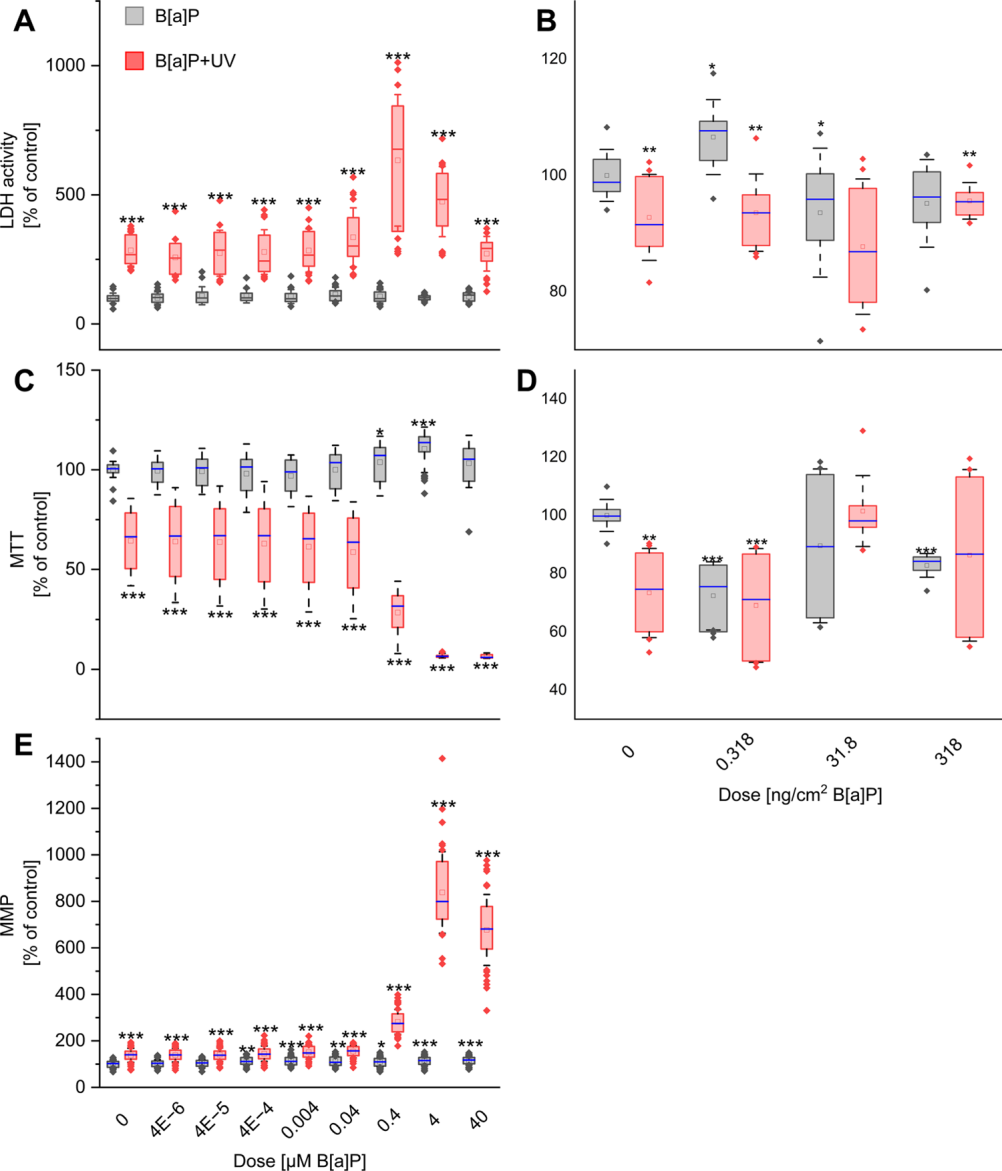
Relative changes of viability in terms of **A**, **B** LDH activity and **C**, **D** the amount of formazan (MTT). **E** MMP assesses the impact on mitochondrial function. Cells (**A**, **C**, **E**) or skin explants **B**, **D** were exposed for 24 h either to B[a]P or UV alone or in combination at the start of exposure. Results represent the mean ± SD. Statistical significance denotes a comparison to control condition without UV irradiation (* = *p* < 0.05; ** = *p* < 0.01; *** = *p* < 0.001). *n* = (keratinocytes: 5 biological replicates, 8 technical/4 technical replicates for LDH; ex vivo: 3 biological replicates, 4 technical replicates) See Supplementary Excel Datasheet for further information

Figure 2 demonstrates that single B[a]P exposure resulted in minimal changes to LDH, MTT, and MMP activity in both keratinocytes and human skin explants. These findings suggest that the B[a]P concentrations used were subcytotoxic, reflecting more realistic environmental and workplace exposure scenarios. Conversely, single UV irradiation elicited significantly stronger effects on these parameters in both models.

We next investigated the impact of combined exposure. In keratinocytes, lower B[a]P concentrations (combined with UV irradiation) did not further decrease viability, which was already compromised by UV alone. However, for all three assays (LDH, MTT and MMP), combined exposure starting at 0.4 µM B[a]P resulted in significantly greater impairment compared to the respective single exposures. The strongest effects were observed at 0.4 µM B[a]P (LDH) or 4 µM B[a]P (MTT, MMP). Notably, at higher B[a]P concentrations, the response became less pronounced, likely due to substantial cytotoxicity (Fig. 2A, C, E).

In contrast to the pronounced effects observed in the cell model, human skin explants exhibited no significant dose-dependent responses to combined treatment compared to UV irradiation alone (Fig. 2B, D).

Our findings suggest that UV irradiation was the primary driver of viability impairment in both models. Combined exposure, however, only further exacerbated this impairment in keratinocytes.

### Combined exposure altered the redox balance only in keratinocytes

To elucidate the potential mechanisms underlying the observed cytotoxicity, particularly with combined exposure, we investigated the cellular redox balance at multiple levels. We began by assessing ROS generation in keratinocytes and its downstream effects on lipid peroxidation, as measured by MDA levels, in both models.

**Fig. 3:**
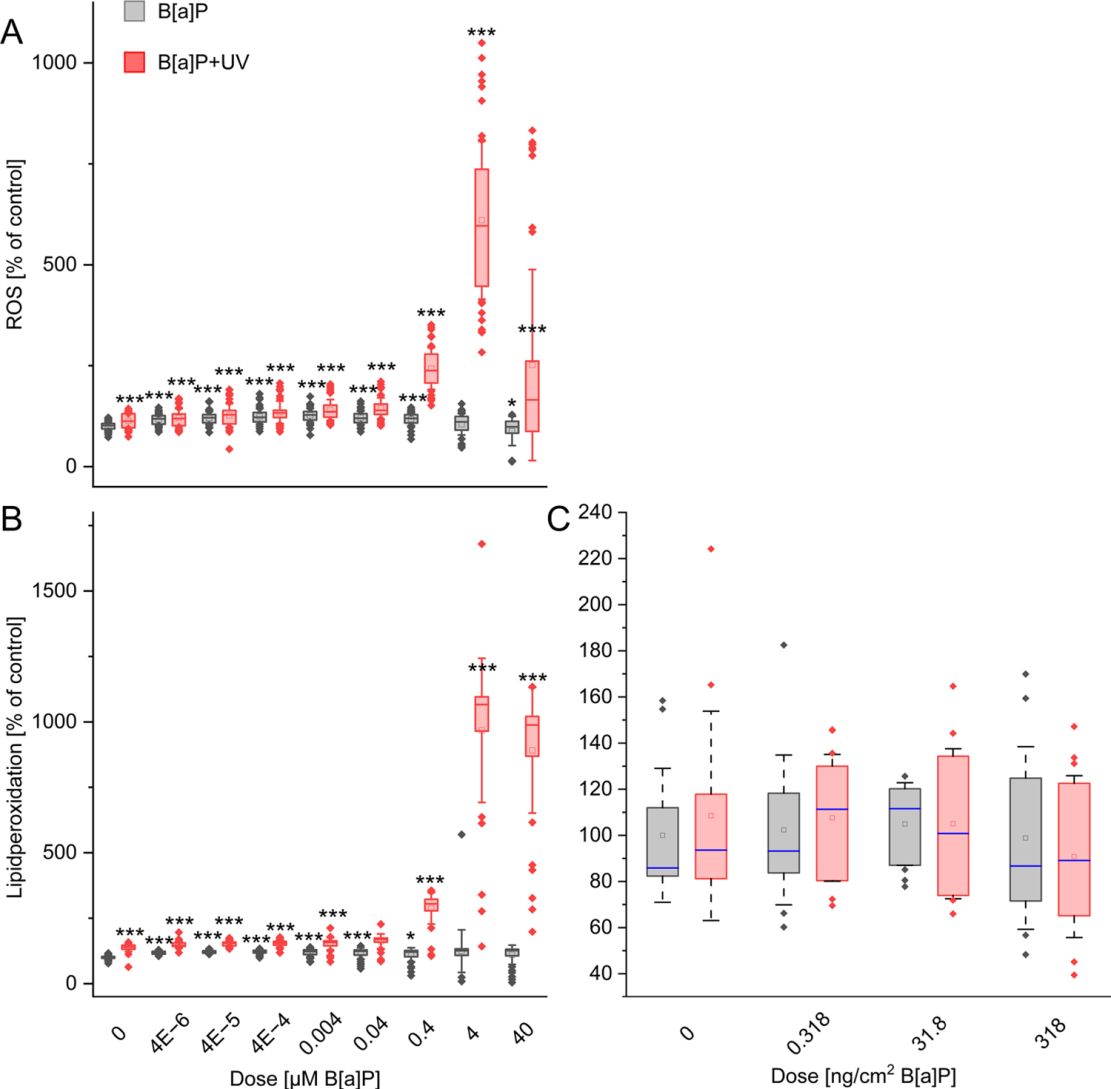
Relative changes of the cellular redox balance by determination of (A) ROS in keratinocytes and lipid peroxidation in (B) keratinocytes and (C) skin explants. Cells or skin explants were exposed for 24 h either to B[a]P or UV alone or in combination at the start of exposure. Results represent the mean ± SD. Statistical significance denotes a comparison to control condition without UV irradiation (* = *p* < 0.05; ** = *p* < 0.01; *** = *p* < 0.001). *n* = (keratinocytes: 5 biological replicates, 8 technical replicates; ex vivo: 3 biological replicates, 4 technical replicates) See Supplementary Excel Datasheet for further information

Single B[a]P exposure caused a moderate increase in ROS levels and triggered lipid peroxidation in keratinocytes (Fig. 3A, B) but induced no significant changes in ex vivo skin (Fig. 3C). Both parameters were more strongly affected by single UV irradiation in keratinocytes, while in ex vivo skin, no significant effect could be detected.

Mirroring the viability results, keratinocytes displayed greater sensitivity to combined exposure. A significant, dose-dependent disruption of the redox balance was observed starting at 0.4 µM B[a]P. In contrast, the combined treatment had no impact on lipid peroxidation in ex vivo skin. Therefore, we concluded that the redox balance was affected in a dose-dependent manner after combined treatment only in keratinocytes.

### Single and combined UV irradiation activated the antioxidant defense system

To get more detailed information, we analyzed the antioxidant defense system in terms of the glutathione system and NQO1 in both models and the Keap1-Nrf2-ARE pathway in keratinocytes.

**Fig. 4:**
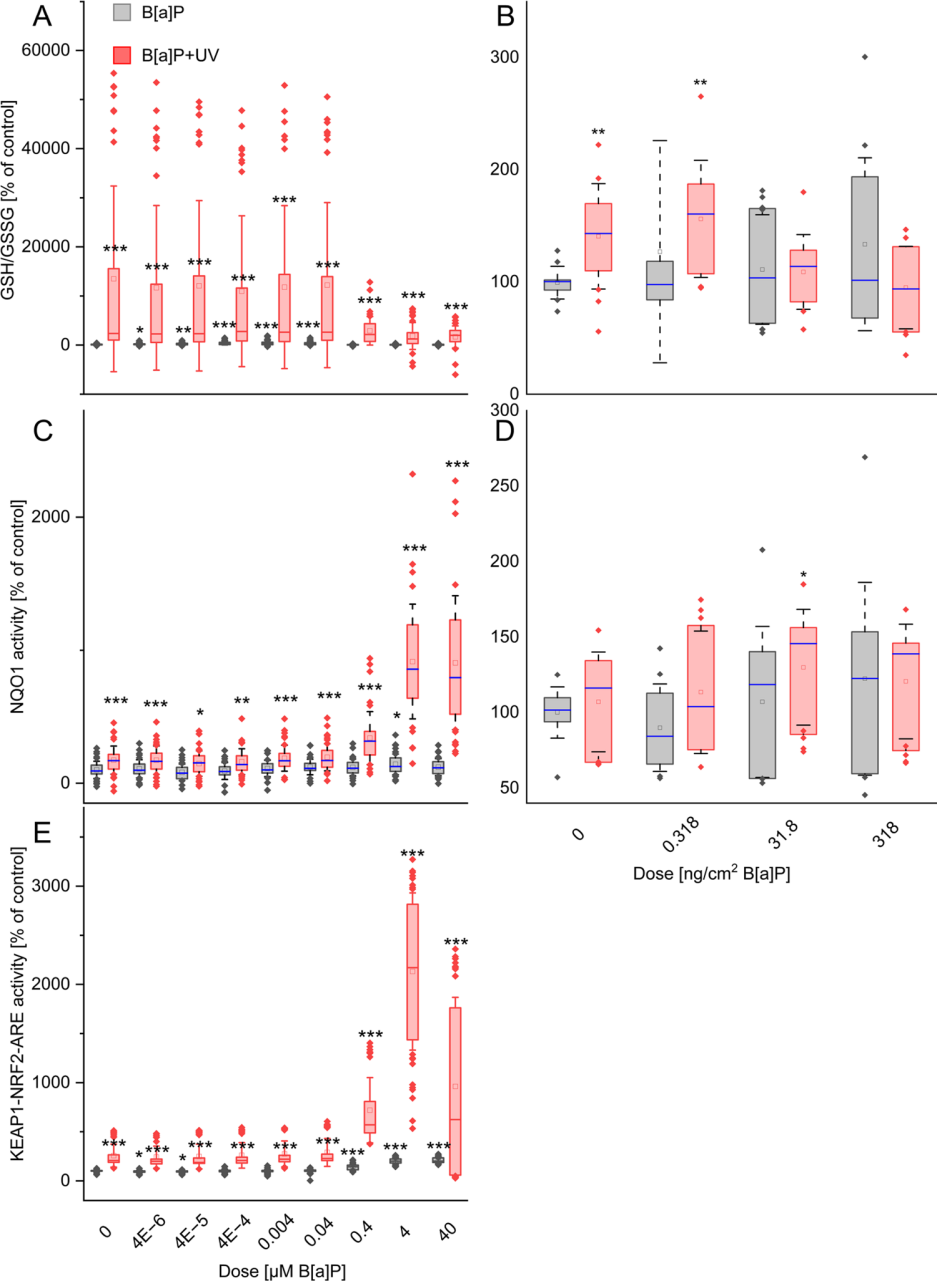
Relative changes in the dose-dependent status of protective antioxidants: **A**, **B** GSH/GSSG ratio, **C**, **D** NQO1, and **E** activation of the Keap1-Nrf2-ARE pathway. **A**,** C**,** E** Cells or **B**, **D** skin explants were exposed for 24 h either to B[a]P or UV alone or in combination at the start of exposure. Results represent the mean ± SD. Statistical significance denotes a comparison to control condition without UV irradiation (* = *p* < 0.05; ** = *p* < 0.01; *** = *p* < 0.001). *n* = (keratinocytes: 5 biological replicates, 8 technical replicates; ex vivo skin: 3 biological replicates, 4 technical replicates) See Supplementary Excel Datasheet for further information

Glutathione (GSH), the most abundant cellular antioxidant, and its ratio to its oxidized form (GSSG) serve as indicators of oxidative stress. Single B[a]P exposure elicited a dose-dependent response in keratinocytes, with the GSH/GSSG ratio initially increasing up to 0.04 µM B[a]P. This suggests an initial activation of antioxidant defenses followed by depletion at higher B[a]P concentrations (Fig. 4A). Conversely, ex vivo skin displayed no significant change in the ratio upon B[a]P treatment (Fig. 4B). In contrast, UV irradiation caused a marked elevation of the GSH/GSSG ratio in both keratinocytes and explants, indicating a robust cellular response to oxidative stress.

Combined B[a]P and UV treatment in keratinocytes revealed an intricate interplay (Fig. 4A). The ratio remained elevated at lower B[a]P concentrations, mirroring the effect of UV alone. However, at higher B[a]P doses, the ratio declined sharply, suggesting potential depletion of GSH stores. Notably, the ratio at the highest combined dose remained significantly higher compared to B[a]P exposure alone. Ex vivo skin exposed to combined treatments exhibited a similar trend (Fig. 4B). The lowest B[a]P dose yielded a ratio comparable to UV exposure alone, suggesting an additive effect. However, higher B[a]P doses resulted in a decrease below the UV-induced baseline, potentially reflecting the exhaustion of antioxidant capacity.

NQO1 activity, a key enzyme in the cellular antioxidant defense system, displayed modest increases following single B[a]P exposure in both keratinocytes and explants (Fig. 4C, D). However, keratinocytes exhibited a slight decrease in activity at the highest B[a]P concentration, potentially reflecting a threshold effect (Fig. 4C). These changes in NQO1 activity generally mirrored the observed trends in the GSH/GSSG ratio. In contrast to B[a]P, single UV irradiation significantly increased NQO1 activity in keratinocytes. Interestingly, combined treatment resulted in a dose-dependent rise in activity compared to either B[a]P or UV alone (Fig. 4C). In ex vivo skin, UV irradiation alone produced a non-significant increase in NQO1 activity (Fig. 4D). However, combined exposure yielded a significant activity increase at all B[a]P concentrations compared to B[a]P exposure alone.

The KEAP1-NRF2-ARE system, a critical pathway in the cellular defense against oxidative and electrophilic stress, was investigated using a luciferase reporter gene assay in KeratinoSens cells (Fig. 4E). Single B[a]P exposure induced a moderate, dose-dependent activation of the system, while single UV irradiation elicited a more pronounced effect. Notably, combined treatment further enhanced the activity, particularly at 0.04 µM B[a]P, surpassing the activation levels observed with either single exposure. However, the highest combined dose reversed this trend, yet the activity remained significantly higher compared to control conditions.

These findings suggest that UV irradiation effectively activated the antioxidant defense system in both keratinocytes and explants, as evidenced by the assessed parameters (GSH/GSSG ratio and NQO1 activity). While combined treatment amplified this response in keratinocytes to a certain extent, its effect on ex vivo skin was significantly lower.

### Model-dependent differences in DNA damaging potency of exposure conditions

To assess the DNA-damaging potential of B[a]P and UV under single and combined exposure conditions, we employed the alkaline comet assay in keratinocytes and evaluated H2AX phosphorylation (γH2AX) levels in both models.

Our comet assay results, using keratinocytes, revealed that single B[a]P exposure did not significantly increase DNA damage, as measured by tail intensity (Fig. S3). Conversely, single UV irradiation caused a marked elevation in tail intensity, indicating substantial DNA strand breaks. Notably, combined B[a]P and UV treatment did not induce further DNA damage beyond that observed with UV alone. While the combined exposure results are of interest, the strong DNA-damaging effect of UV irradiation alone necessitates careful interpretation.

**Fig. 5:**
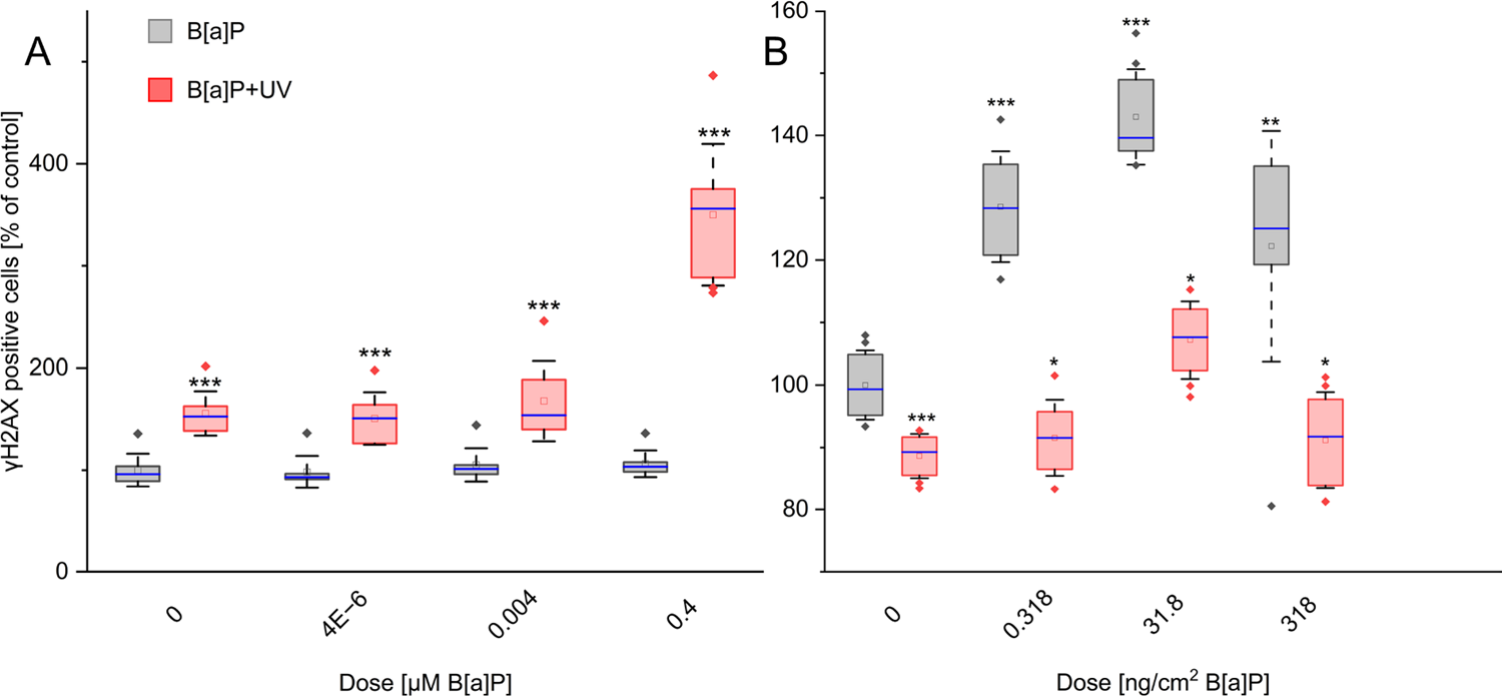
Changes in DNA damage levels: **A**, **B** the percentage of γH2AX-positive cells as compared to control in both models.. Results represent the mean ± SD. Statistical significance denotes a comparison to the same condition without UV irradiation (* = *p* < 0.05; ** = *p* < 0.01; *** = *p* < 0.001); *n* = γH2AX; 2 biological replicates, 4 technical replicates in both models See Supplementary Excel Datasheet for further information

Consistent with the comet assay, γH2AX immunofluorescence demonstrated a negligible effect of single B[a]P exposure on DNA double-strand breaks in keratinocytes (Fig. 5A). In contrast, UV irradiation alone significantly induced γH2AX foci. Interestingly, combined treatment further enhanced γH2AX induction at the highest B[a]P concentration, suggesting a potential additive effect on DNA double-strand break formation (Fig. 5A).

Ex vivo skin explants displayed a distinct response compared to keratinocytes (Fig. 5B). Here, single B[a]P exposure triggered a dose-dependent increase in γH2AX phosphorylation up to 31.8 ng/cm^2^ B[a]P, followed by a decline at higher concentrations. Notably, even after this decline, γH2AX levels remained elevated compared to controls. Conversely, single UV irradiation resulted in a decrease in γH2AX levels, potentially reflecting activation of DNA repair pathways. Interestingly, combined treatment with B[a]P and UV induced γH2AX phosphorylation, but to a significantly lower extent compared to B[a]P exposure alone.

These findings highlight distinct DNA damage responses between the two models. Keratinocytes exhibited high sensitivity to UV irradiation, with minimal additional DNA damage upon combined exposure. In contrast, ex vivo skin displayed a dose-dependent increase in DNA damage following single B[a]P exposure, suggesting a potentially compromised DNA repair capacity. Furthermore, UV irradiation alone appeared to reduce γH2AX levels in ex vivo skin, which might counteract the genotoxic effects of additional B[a]P exposure.

### Sensitivity ranking across endpoints using benchmark analysis in keratinocytes

Our intention was to quantitatively compare the endpoint sensitivity across treatment conditions and between the models by employing a benchmark approach. However, due to the non-monotonic dose–response observed in ex vivo skin, this approach was only applicable to the data generated from keratinocytes. Keratinocytes exhibited a pronounced increase in B[a]P response following UV irradiation. To explore this further, we employed a benchmark dose (BMD) approach to rank endpoints based on their sensitivity to combined B[a]P and UV exposure. DNA damage (γH2AX) emerged as the most sensitive endpoint, exhibiting the earliest response. This suggests a rapid and significant increase in DNA double-strand breaks upon combined treatment. Notably, viability/cytotoxicity endpoints displayed a lower level of sensitivity, indicating a delayed impact on cell survival. Interestingly, the glutathione system appeared as the most robust endpoint, with the least impact observed upon combined treatment (additional information is provided in the Supplementary Data; Fig. S3, Tab. S1).

### B[a]P uptake and synergy

Results of B[a]P uptake and synergy calculations are presented in the Supplementary Data (Fig. S2, Tabl. S2).

## Discussion

UV irradiation and PAHs, exemplified by B[a]P, are established risk factors for skin cancer. While their independent contributions to carcinogenesis are well-documented, the potential for synergistic interactions remains controversial (von Koschembahr et al. 2020b; Weistenhofer et al. 2022). To address this knowledge gap, we investigated the combined impact of UV and B[a]P on cellular responses relevant to skin cancer development, focusing on antioxidant defenses and DNA damage, key players in this process. We employed both in vitro keratinocyte culture and a human ex vivo skin model to gain a deeper understanding of these combined effects. While in vitro cell line cultures, such as the keratinocytes used here, offer advantages in terms of availability, reproducibility, and ease of manipulation for detailed cellular and molecular analyses, including genetic modification as demonstrated with our KeratinoSens cell line, they lack the complex architecture and intercellular interactions present in intact skin tissue, which includes a variety of cell types in contrast to the single cell type present in the two-dimensional keratinocyte culture. Ex vivo skin, on the other hand, retains a structure similar to live skin, providing a more physiologically relevant context. However, ex vivo skin has limitations, including limited availability and assay restrictions. The inherent differences in robustness and complexity between these two models are likely of particular significance to our study.

Uptake and metabolism are critical determinants of B[a]P toxicity. To assess these processes, we employed a simplified technique in keratinocytes following B[a]P exposure. Although not the standard method, this provided us with preliminary data on the cellular response (Verma et al. 2012). While combined B[a]P and UV treatment showed a trend towards increased B[a]P uptake compared to single exposure, the effects on 3-OH B[a]P and B[a]P-tetrol formation differed. 3-OH B[a]P levels were notably elevated, whereas B[a]P-tetrol levels increased only modestly, despite statistical significance. This observed trend for B[a]P-tetrol is in line with findings reported in the literature, though further investigation is warranted (Bourgart et al. 2019a, 2019b; Hopf et al. 2018). Although our investigations were conducted solely in keratinocytes, increased B[a]P accumulation following UV irradiation has also been reported in human ex vivo skin, suggesting that B[a]P uptake and metabolism likely occurred in our skin model as well (Bourgart et al. 2019b). Notably, B[a]P-tetrol formation is of interest as its precursors are DNA-damaging diol-epoxides, primarily generated by CYP1 A1 (Baird et al. 2005; Xia et al. 2015). We observed a synergistic effect on CYP activation at higher combined B[a]P and UV doses compared to single exposure in both models. However, the data on combined treatment’s effect on CYP gene expression in ex vivo skin is inconclusive, potentially due to limited dose ranges (von Koschembahr et al. 2020a, 2018). Nevertheless, our observed synergy is plausible as both B[a]P and UVB independently induce CYP 1 A1 and 1B1 gene expression (Costa et al. 2010; Katiyar et al. 2000; Nair et al. 2009; Villard et al. 2002; von Koschembahr et al. 2020a, 2018).

To gain a comprehensive understanding of potential genotoxicity, we employed a multi-assay approach that evaluated cytotoxicity and viability alongside genotoxicity endpoints. This approach provides a more holistic picture compared to studies solely focused on genotoxicity. UV irradiation exhibited greater cytotoxicity compared to B[a]P exposure alone especially in keratinocytes. Consistent with previous reports, the combined treatment displayed a synergistic cytotoxic effect (Crallan et al. 2005; von Koschembahr et al. 2020b; Wang et al. 2007). This effect was observed in keratinocytes at higher B[a]P concentrations (0.4 µM and above). This dose-dependent response suggests a transition to an adverse cellular response beyond this threshold. However, ex vivo skin exhibited significantly higher resistance to the combined treatment compared to keratinocytes, highlighting potential limitations in generalizability to in vivo settings.

A possible explanation for the transition is oxidative stress. B[a]P can be converted to reactive intermediates by photochemical processes (Fu et al. 2012; Yu 2002), potentially amplifying B[a]P-induced phototoxicity and oxidative stress at specific B[a]P concentrations. This could overwhelm cellular antioxidant defenses, leading to cytotoxicity and DNA damage (Masutin et al. 2024; Verma et al. 2017). Glutathione, NQO1, and Keap1-Nrf2-ARE are important factors in the response to oxidative stress. Reduced glutathione also participates in the detoxification of B[a]P (Jernstrom et al. 1989). Within our study, NQO1 emerges as a particularly noteworthy player due to its ability to neutralize B[a]P quinones, potent inducers of oxidative stress. NQO1 is a highly inducible enzyme tightly regulated by the Keap1/Nrf2/ARE pathway. This pathway hinges on the oxidative modification of Keap1’s cysteine residues by electrophilic agents, including B[a]P quinones and ROS (Wondrak et al. 2008). Interestingly, UV exposure alone increased antioxidant parameters in both models, but only keratinocytes displayed elevated ROS and MDA levels. This upregulation of protective mechanisms by UV has been observed in other skin cell studies (Hirota et al. 2005; Tang et al. 2018). While both models likely exhibited this phenomenon, the ex vivo skin displayed a more robust antioxidant response that effectively neutralized oxidative stress and restored cellular redox balance. Additionally, the BMD analysis suggests a particularly strong response of the glutathione system in keratinocytes, further highlighting its resilience against combined B[a]P and UV exposure.

DNA damage and repair are fundamental processes in tumor development. Our study revealed distinct responses to DNA damage between the in vitro and ex vivo models. Keratinocytes displayed a marked increase in DNA damage following both single and combined B[a]P and UV exposure. Conversely, skin explants exhibited a reduction in DNA damage after single UV exposure, and even combined exposure did not reach the levels observed with single B[a]P exposure. This potentially reflects a more efficient DNA repair mechanism in ex vivo skin, leading to lower levels of detectable damage after 24 h. It is important to note that these findings represent a single time point. Further research is necessary to understand the long-term consequences of combined UV and B[a]P exposure on DNA damage in skin explants. Notably, the divergent DNA damage responses observed in the models, coupled with the limited oxidative stress observed in ex vivo skin, align with the lack of evidence for a synergistic carcinogenic effect of UV and PAHs in epidemiological studies (Weistenhofer et al. 2022).

## Conclusion

Our study underscores the importance of model selection when evaluating synergistic effects of combined UV and B[a]P exposure. Keratinocytes, due to their simplified nature, exhibited a more pronounced response compared to the more robust and complex ex vivo skin explants. This difference was evident in distinct patterns of oxidative balance and DNA damage. Keratinocytes displayed a synergistic and adverse response at a B[a]P concentration of 0.4 µM. Conversely, in skin explants, a dose-dependent response was observed only for a limited number of parameters. Notably, UV irradiation alone induced an antioxidant defense response in both models. However, this response was sufficient to mitigate oxidative stress only in the ex vivo skin explants. It is likely that the skin explants exhibited an improved DNA repair capacity, as evidenced by a reduction in γH2AX concentration after UV irradiation, which effectively counteracted B[a]P-induced damage.

## Supplementary Information

Below is the link to the electronic supplementary material.

Supplementary file1 (DOCX 835 KB)

Supplementary file2 (XLSX 4575 KB)

## Data Availability

Data are available from the corresponding author upon request.
