# Supplementary material for: Benzo[a]pyrene and UV light co-exposure: differential effects on oxidative stress and genotoxicity in human keratinocytes and ex vivo skin
Source: Arch Toxicol. 2025 Jul 1;99(10):4215–26. doi: 10.1007/s00204-025-04098-w (PMC12454581; doi:10.1007/s00204-025-04098-w)
Supplement: Supplementary file 1 — Supplementary file1 (DOCX 835 KB) [file 204_2025_4098_MOESM1_ESM.docx]

Supplementary Information

Benzo[a]pyrene and UV light co-exposure: Differential effects on oxidative stress and genotoxicity in human keratinocytes and ex vivo skin

Christian Kersch, Viktor Masutin, Rasha Alsaleh, Simone Schmitz-Spanke

Institute and Outpatient Clinic of Occupational, Social, and Environmental Medicine, Friedrich-Alexander-University of Erlangen-Nuremberg, Henkestr. 9–11, 91054 Erlangen, Germany

Christian.kersch@fau.de; viktor.masutin@fau.de; rasha.alsaleh@fau.de; simone.schmitz-spanke@fau.de

Corresponding Author:

Simone Schmitz-Spanke

simone.schmitz-spanke@fau.de

Institute and Outpatient Clinic of Occupational, Social, and Environmental Medicine, University of Erlangen-Nuremberg, Henkestr. 9-11, 91054 Erlangen, Germany

Phone: +49 09131/85-22255

ORCID: 0000-0002-0416-8236

**Table of Contents:**

Supplementary Material and Methods

Supplementary Results

**Supplementary Methods**

**Cell culture**

KeratinoSens cells (obtained from Givaudan, Vernier, Switzerland) grown in Dulbecco's Modified Eagle Medium (DMEM) supplemented with 1 g/L glucose, 10% fetal calf serum (FCS), 1% G418, and 2% glutamine until reaching 80% confluence before being passaged. For experiments, cells were seeded in DMEM supplemented with 1 g/L glucose, 1% FCS, and 2% glutamine (called here exposure medium). A consistent seeding density of 100,000 cells/mL was employed across all plate formats (96-well, 6-well, etc.). In the case of 96-well plates, each well received a 100 µL aliquot of the cell suspension, resulting in a seeding of 10,000 cells per well. Cells were cultured at 37 °C in a humidified incubator with 5% CO2 and incubated for 24 h before exposure.

**Skin**

Written informed consent was obtained from all skin donors. The study was approved by the Ethics Commission of the Friedrich-Alexander-University Erlangen-Nürnberg (Ethics Approval #261_14B). Freshly excised human abdominal skin, obtained anonymously from the Department of Plastic and Hand Surgery at the Friedrich-Alexander University, was used for these experiments

**UV dose selection**

According to the Deutscher Wetterdienst in 2018 Germany had an average of 1207 kWh/m^2^ solar radiation measured on a flat surface with 2015.4 hours of sunlight. With 5 % of UV-light in solar radiation reaching the earth surface 3.5J/cm^2^ correspond to ca. 20 minutes of sunlight (IARC 2012). For our skin model we chose to double the UV dose due to the model being more robust.

**Cell exposure**

Cells were exposed to B[a]P concentrations ranging from 0.000004 - 40 µM.

Following B[a]P application, one plate was immediately placed in the incubator for control conditions. The other plate was exposed to UV light (3.5 J, 95% UVA, 5% UVB).

**Skin exposure**

In the cell culture experiments, keratinocytes were exposed to B[a]P at a concentration of 4 µM in 200 µL of medium within 96-well plates. Based on the molecular weight of B[a]P (252.33 g/mol), this equates to 2.02 x 10⁻⁷ g B[a]P per well. For the ex vivo skin model, skin explants were prepared using tissue punches with a diameter of 0.9 cm, resulting in a surface area of 0.636 cm². B[a]P was topically applied to the skin explants at a concentration of 318 ng/cm² (2.02 x 10^-7^ g in 10 µL acetone), which was calculated to be equivalent to the 4 µM B[a]P concentration used in the cell culture experiments. To assess dose-dependent effects in the skin explant model, additional B[a]P doses were used, corresponding to 0.004 µM and 0.4 µM B[a]P in the cell culture system (2.02 x 10^-10^ and 2.02 x 10^-8^ g in 10 µL acetone respectively).

**Preparation and exposure of skin explants**

Only the subcutaneous fat was removed, using a full thickness model. Skin integrity was visually assessed prior to proceeding. Nine-millimeter punch biopsies were then extracted and placed in six-well plates. The biopsies were maintained at 37 °C in a humidified incubator containing 5% carbon dioxide using DMEM/F-12 medium (cc-Pro, Oberdorla) supplemented with 1% penicillin/streptomycin and 4% bovine serum albumin (complete medium). Each well of a 6-well plate received 500 µL of complete medium. Skin punches were then placed on top of the medium.

Benzo[a]pyrene (B[a]P) was topically applied to the skin at concentrations of 0.318, 31.8, and 318 ng/cm². This involved applying 10 µL of a B[a]P solution prepared in acetone to the skin surface. The acetone was allowed to evaporate for 10 minutes.

Following B[a]P application, one plate was immediately placed in the incubator for control conditions. The other plate was exposed to UV light (7J, 95% UVA, 5% UVB).

After 24 hours of incubation/exposure, the medium and skin were collected separately. The medium was either used for immediate analysis or frozen for later use. The skin punches were weighed and then cut into 8 pieces for further processing.

In both exposure conditions, samples were collected 24 h after the beginning of the treatment. After the exposure, if required, the skin samples were minced with a scalpel and homogenized with a Bead Genie at 2400 rpm for 5 minutes in 500 mL PBS. Unless otherwise specified, experiments utilized 50 µL of homogenate per well (96-well plate). For these experiments, solution concentrations were adjusted to achieve final concentrations in the assays that mirrored those used for keratinocytes. Explants from eight different donors (5 female, 3 male) were employed, with samples prepared in triplicate and analyzed with four technical replicates. Due to limitations of the ex vivo skin model, some assays were not compatible and could not be performed.

**Assessment of B[a]P uptake and metabolism in dependence of time, B[a]P dose and UV**

Because of the fluorescence of B[a]P and some of its metabolites, B[a]P uptake and metabolic activation was monitored in keratinocytes, as previously described (Verma et al. 2012) with modifications. Keratinocytes were exposed to a range of B[a]P concentrations (0.000004 - 40 µM) for up to 48 hours. A multimode microplate reader (Varioscan LUX 40, Life Technologies by Thermo Fisher Scientific, Waltham, USA) was used to measure fluorescence emission of B[a]P and its metabolites. Specific excitation and emission wavelengths were used to monitor B[a]P (λ(ex/em) 296/405 nm), B[a]P-tetrol (λ(ex/em) 345/389 nm), and 3-OH B[a]P (λ(ex/em) 365/430 nm)(Bourgart et al. 2019). Experiment was performed twice with eight technical replicates per analysis. The 40 µM dose is depicted in the graph.

**Assessment of cytotoxicity, metabolic activity, and mitochondrial function**

For the MTT assay with keratinocytes, 20 µL of MTT solution (5 mg/mL) was added 2 hours before the end of exposure, resulting in a final concentration of 0.13 mM. Following incubation, the supernatant was removed and replaced with 100 µL of solubilization solution (0.6% glacial acetic acid and 10% SDS in DMSO). The plates were then incubated for 10 minutes with agitation. Finally, the absorbance was measured at 600 nm.

For skin explants, 1 mL of MTT solution (0.5 mg/mL) was added and incubated for 2 hours. The explants were then shredded and transferred to 1 mL of solubilization solution. The skin samples were minced with a scalpel and homogenized using a Bead Genie at 2400 rpm for 5 minutes. After centrifugation at 14,000 rpm for 5 minutes, 100 µL of the resulting solution was transferred to a clear 96-well plate and the absorbance measured at 600 nm..

MMP was analyzed by using rhodamin 123, a fluorescent mitochondrial membrane potential indicator (Rh123, Life Technologies by Thermo Fisher Scientific, Waltham, USA). After exposure, the cells were washed with PBS. Rh123 (1 µM) was added for 30 min at 37 °C, 5% CO2. After washing twice, the fluorescence measurement was performed in 200 µL PBS/well at λ(ex/em) 485/535 nm (Rh123).

A modified LDH assay, based on a protocol from opsdiagnostics.com, was employed to assess LDH activity. The assay involved mixing 50 µL of cell culture supernatant with 50 µL of a reaction solution in a 96-well plate. The reaction solution comprised 200 mM Tris-HCl (pH 8.0), 50 mM sodium lactate, and a PMS/INT/NAD^+^ solution. This latter solution was prepared by combining 100 µL of 29 mM PMS (phenazine methosulfate) in water, 100 µL of 65 mM INT (2-(p-iodophenyl)-3-(p-nitrophenyl)-5-phenyltetrazolium chloride) in DMSO, and 2.3 mL of 5 mM NAD^+^ (nicotinamide adenine dinucleotide) in water. After a 5-minute incubation at room temperature, the absorbance was measured at 490 nm.

**Assessment of the oxidative stress**

For further information on the Glutathione Assay see the publication from Pink *et al.*(Pink et al. 2017)

**Comet assay**

For the comet assay under alkaline conditions, the cells were directly washed twice with PBS and trypsinized. After preparing a cell suspension of 250,000 cells/mL, 20 µL were added to 140 µL of 1% low-melting agarose (38 °C).

Of this mixture, 20 µl were pipetted onto a precoated slide. Positive-control cells were treated with 50 µM H2O2 for 5 min. Cells were lysed (2.5 M NaCl, 0.1 M EDTA, 10mM Tris, 1% Triton X-100) on ice for one hour in the dark. After lysis, slides were placed in 4 °C alkaline electrophoresis buffer (0.3 M NaOH, 1mM EDTA) for 40 min. After that, electrophoresis was run for 25 min at 4 °C (1 V/cm, 300 mA). Gels were washed twice with 0.04 M Tris for 5 min and once with water for 3 min before being stored in the dark to completely dry. They were stained with SYBR®-Green nucleic acid stain (Invitrogen by Thermo Fisher Scientific, Waltham, USA) in 1:10000 dilution. Gels were examined by using the comet-assay IV software (Perspective Instruments, UK) and a Leica microscope attached to a CDD camera. Values of the tail intensity, counting 100 comets, were automatically calculated by the software (Di Bucchianico et al. 2018; Wohlfahrt et al. 2024). The assay was carried out using three biological replicates.

**Calculation of synergy**

To assess synergy, a basic additive model was employed. The effect of the combined B[a]P and UV exposure was compared to the sum of the individual effects of B[a]P (0.4 µM) and 3.5J/cm^2^ UV exposure. This dose of B[a]P was chosen because it corresponded to the initial observation of adverse effects in most measured parameters. A positive difference between the combined and individual effects was considered synergistic, while a negative difference indicated antagonism (Greco et al. 1996; Roell et al. 2017).

**Supplementary Results**

**Preliminary experiments to determine the B[a]P concentrations.**

Cell viability was assessed using the Neutral Red assay to determine dose-dependent effects. Previous work in our laboratory, utilizing different cell models, suggested that B[a]P concentrations of 0.05 µM or 0.5 µM and above could elicit initial adverse responses (Verma et al. 2013; Wohlfahrt et al. 2024). Preliminary experiments investigated the impact of additional UV irradiation on B[a]P-induced effects. To encompass the adaptive response range and characterize the transition to adverse effects, eight B[a]P concentrations, with and without prior UV irradiation, were employed in these initial studies. While single B[a]P exposure elicited minimal effects, combined exposure (B[a]P with UV) resulted in impaired cell viability at B[a]P concentrations ≥0.4 µM. Consequently, these same eight B[a]P concentrations were used in the present study. This approach was adopted to establish a comprehensive dose-response curve, initiating within the non-adverse range, as the potential for other toxicological endpoints to exhibit adverse responses at lower B[a]P concentrations was not yet established.

For the assay, neutral red was added 22 hours post-treatment to a final concentration of 63 µM for 2 hours. Following a PBS wash, cells were lysed with 100 µL of a solution containing 1% acetic acid in 50% ethanol. After a 10-minute incubation on an orbital shaker, absorbance was measured at 540 nm, with a reading at 645 nm used for background correction.





Figure S1: Relative changes of the dose-dependent neutral red uptake for the estimation of cell viability/cytotoxicity in keratinocytes. Results represent the mean ± SD. Statistical significance denotes a comparison to control condition without UV irradiation (* = p < 0.05; ** = p < 0.01; *** = p < 0.001). (5 biological replicates, 8 technical replicates)

**Time-dependent increase in fluorescence intensity of B[a]P, 3-OH B[a]P, and B[a]P-tetrol and the effect of UV irradiation in keratinocytes**

B[a]P is biologically inert and its toxicity depends upon the ability of a given tissue to take it up and to form active metabolites. To assess this process, we investigated the uptake and formation of two metabolites (B[a]P-tetrol and 3-OH B[a]P) by measuring their fluorescence in keratinocytes and how UV irradiation affects this.
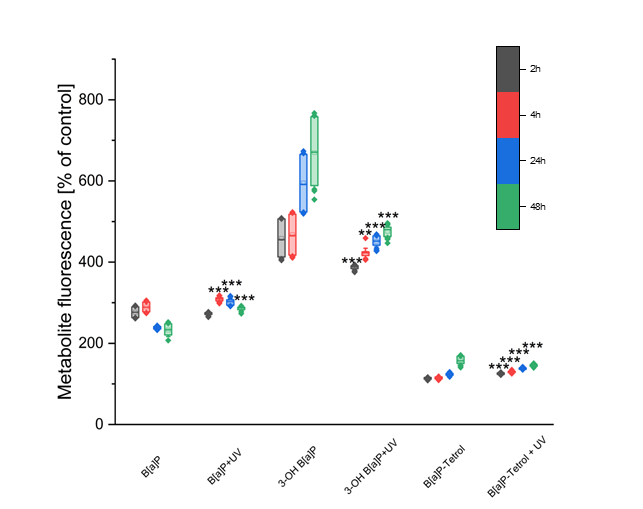


Fig. S2: Time-dependent increase in fluorescence intensity of B[a]P, 3-OH B[a]P, and B[a]P-tetrol in the absence and presence of UV irradiation in keratinocytes. Cells were exposed to 40 µM B[a]P and fluorescence of B[a]P and its metabolites was monitored using a multimode microplate reader. Results represent the mean ± SD. Statistical significance denotes a comparison to the same condition without UV irradiation. (* = p < 0.05; ** = p < 0.01; *** = p < 0.001). n= (2 biological replicates, 8 technical replicates)

The time-dependent increase of the fluorescence intensity of 3-OH B[a]P and B[a]P-tetrol indicated the uptake and metabolic activation of B[a]P in keratinocytes (Fig. S1).

B[a]P incubation followed by UV irradiation resulted in a time-dependent increase in B[a]P fluorescence intensity. Conversely, 3-OH B[a]P fluorescence was significantly lower after UV irradiation compared to non-irradiated controls. Interestingly, B[a]P-tetrol fluorescence exhibited a slight but significant increase following UV exposure.

**DNA strand breaks are primarily induced by single UV irradiation in keratinocytes**

*

*

*Figure S3: Induction of DNA strand breaks in keratinocytes was investigated using the alkaline comet assay. Cells for comet assay were exposed for 2 h either to B[a]P/ alone or with simultaneous UV irradiation at the start of exposure. Results are expressed as % DNA in tail and represent the mean ± SD. Statistical significance denotes a comparison to the same condition without UV irradiation (* = p < 0.05; ** = p < 0.01; *** = p < 0.001); n= 3 biological replicates. See Supplementary Excel Datasheet for further information.*

**Benchmark dose (BMD) calculation for measured endpoints in keratinocytes**





Fig. S4: BMD (Benchmark Dose) values for in vitro assays are presented with corresponding 95% confidence intervals calculated using Proast software. The model with the lowest Akaike Information Criterion (AIC) was chosen for each endpoint. The BMD approach was used to rank the endpoints based on their sensitivity to combined B[a]P and UV exposure. Detailed values are provided in Table S1.

Table S1: BMDs for endpoints in keratinocytes with corresponding BMDLs, BMDUs and chosen model.

| Parameter | BMDL | BMR | BMDU | Modell |
| --- | --- | --- | --- | --- |
| γH2AX | 0.0006 | 0.0006495 | 0.0002 | Hill m3- |
| LDH | 4.11E-05 | 0.004827 | 0.19 | Expon. m3- |
| CYP | 0.00165 | 0.005397 | 0.0139 | Hill m5- |
| ROS | 0.000261 | 0.005595 | 0.0276 | Expon. m5- |
| NQO1 | 0.00234 | 0.01032 | 0.0537 | Expon. m5- |
| Keap1-NRF2-ARE | 0.00525 | 0.01322 | 0.0341 | Expon. m5- |
| MMP | 0.00754 | 0.03396 | 0.207 | Expon. m5- |
| Lipidperoxidation | 0.0275 | 0.04694 | 0.14 | Expon. m5- |
| MTT | 0.00048 | 0.04802 | 1.05 | Expon. m3- |
| Glutathione | 0.0111 | 0.2094 | 0.22 | Expon. m5- |

**Synergy calculation**

Table S2: Synergy calculations for 0.4 µM B[a]P and 3.5J/cm^2^ UV irradiation as combined dose. The sum of the relative changes in the single exposures is compared to the combined exposure

| Parameter | Sum of rel. change compared to control after 0.4 µM B[a]P / UV | Rel. Change compared to control after combined exposure | Difference | Evaluation |
| --- | --- | --- | --- | --- |
| MTT | 31.90502737 | 71.51572305 | 39.61069568 | Synergistic |
| MMP | 47.19651487 | 181.6801158 | 134.483601 | Synergistic |
| CYP450 | 76.96351215 | 224.0747739 | 147.1112617 | Synergistic |
| LDH | 191.471449 | 533.678623 | 342.207174 | Synergistic |
| NQO1 | 91.94417847 | 238.9348447 | 146.9906662 | Synergistic |
| Lipidperoxidation | 44.25495997 | 181.9243657 | 137.6694057 | Synergistic |
| Keap1-NRF2-ARE | 185.2259825 | 585.5084377 | 400.2824552 | Synergistic |
| ROS | 30.28544402 | 142.9068021 | 112.621358 | Synergistic |
| Comet | 106.1351208 | 85.8651418 | -20.26997898 | Antagonistic |
| γH2AX | 61.91456016 | 249.9592128 | 188.0446527 | Synergistic |
| Glutathione | 13332.90254 | 2806.982028 | -10525.92051 | Antagonistic |

**References**Bourgart E, Barbeau D, Marques M, et al. (2019) A realistic human skin model to study benzo[a]pyrene cutaneous absorption in order to determine the most relevant biomarker for carcinogenic exposure. Arch Toxicol 93(1):81-93 doi:10.1007/s00204-018-2329-2

Di Bucchianico S, Gliga AR, Akerlund E, et al. (2018) Calcium-dependent cyto- and genotoxicity of nickel metal and nickel oxide nanoparticles in human lung cells. Particle and fibre toxicology 15(1):32 doi:10.1186/s12989-018-0268-y

Greco WR, Faessel H, Levasseur L (1996) The search for cytotoxic synergy between anticancer agents: a case of Dorothy and the ruby slippers? J Natl Cancer Inst 88(11):699-700 doi:10.1093/jnci/88.11.699

IARC (2012) Solar and ultraviolet radiation. IARC Monographs on the Evaluation of Carcinogenic Risks to Humans, No 100D

Pink M, Verma N, Zerries A, Schmitz-Spanke S (2017) Dose-Dependent Response to 3-Nitrobenzanthrone Exposure in Human Urothelial Cancer Cells. Chemical research in toxicology 30(10):1855-1864 doi:10.1021/acs.chemrestox.7b00174

Roell KR, Reif DM, Motsinger-Reif AA (2017) An Introduction to Terminology and Methodology of Chemical Synergy-Perspectives from Across Disciplines. Front Pharmacol 8:158 doi:10.3389/fphar.2017.00158

Verma N, Pink M, Petrat F, Rettenmeier AW, Schmitz-Spanke S (2012) Exposure of primary porcine urothelial cells to benzo(a)pyrene: in vitro uptake, intracellular concentration, and biological response. Arch Toxicol 86(12):1861-71 doi:10.1007/s00204-012-0899-y

Verma N, Pink M, Rettenmeier AW, Schmitz-Spanke S (2013) Benzo[a]pyrene-mediated toxicity in primary pig bladder epithelial cells: a proteomic approach. Journal of proteomics 85:53-64 doi:10.1016/j.jprot.2013.04.016

Wohlfahrt J, Verma N, Alsaleh R, Kersch C, Schmitz-Spanke S (2024) A pilot study exploring time- and dose-dependent DNA damage and chromosomal instability caused by benzo[a]pyrene in two urothelial cell types. Mutation research 828:111855 doi:10.1016/j.mrfmmm.2024.111855
